# Inhibition of *de novo* pyrimidine synthesis augments Gemcitabine induced growth inhibition in an immunocompetent model of pancreatic cancer

**DOI:** 10.7150/ijbs.60473

**Published:** 2021-06-01

**Authors:** Thuy Phan, Vu H. Nguyen, Ralf Buettner, Corey Morales, Lifeng Yang, Paul Wong, Weiman Tsai, Marcela d'Alincourt Salazar, Ziv Gil, Don J Diamond, Joshua D. Rabinowitz, Steven Rosen, Laleh G. Melstrom

**Affiliations:** 1 Department of Surgery, City of Hope National Medical Center, Duarte, CA 91010.; 2 Department of Hematology, City of Hope National Medical Center, Duarte, CA 91010.; 3 Lewis Sigler Institute for Integrative Genomics and Department of Chemistry, Princeton University, Princeton, NJ 08544.; 4 Rambam Medical Center, Israel.

**Keywords:** pancreatic cancer, leflunomide, *de novo* pyrimidine synthesis

## Abstract

Leflunomide (Lef) is an agent used in autoimmune disorders that interferes with DNA synthesis. *De Novo* pyrimidine synthesis is a mechanism of Gemcitabine (Gem) resistance in pancreatic cancer. This study aims to assess the efficacy and changes in the tumor microenvironment of Lef monotherapy and in combination with Gem, in a syngeneic mouse model of pancreatic cancer.

**Methods:** MTS proliferation assays were conducted to assess growth inhibition by Gem (0-20 nM), Lef (0-40 uM) and Gem+Lef in KPC (KrasLSL.G12D/+;p53R172H/+; PdxCretg/+) cells *in vitro*. An *in vivo* heterotopic KPC model was used and cohorts were treated with: PBS (control), Gem (75 mg/kg/q3d), Lef (40 mg/kg/d), or Gem+Lef. At d28 post-treatment, tumor burden, proliferation index (Ki67), and vascularity (CD31) were measured. Changes in the frequency of peripheral and intratumoral immune cell subsets were evaluated via FACS. Liquid chromatography-mass spectrometry was used for metabolomics profiling.

**Results:** Lef inhibits KPC cell growth and synergizes with Gem *in vitro* (P<0.05; Combination Index 0.44 (<1 indicates synergy). *In vivo*, Lef alone and in combination with Gem delays KPC tumor progression (P<0.001). CTLA-4+T cells are also significantly decreased in tumors treated with Lef, Gem or in combination (Gem+Lef) compared to controls (P<0.05). Combination therapy also decreased the Ki67 and vascularity (P<0.01). Leflunomide inhibits *de novo* pyrimidine synthesis both *in vitro* (p<0.0001) and *in vivo* (p<0.05).

**Conclusions:** In this study, we demonstrated that Gem+Lef inhibits pancreatic cancer growth, decrease T cell exhaustion, vascularity and as proof of principle inhibits *de novo* pyrimidine synthesis. Further characterization of changes in adaptive immunity are necessary to characterize the mechanism of tumor growth inhibition and facilitate translation to a clinical trial.

## Introduction

Pancreatic ductal adenocarcinoma (PDAC) is frequently an incurable disease with less than 9% five year survival 1. Due to the lack of symptoms the disease has often metastasized by the time of diagnosis. Less than 20% of patients are candidates for tumor resection 2. Thus most patients undergo palliative systemic therapy. Therapeutic options for patients with metastatic PDAC that have either become resistant to first line chemotherapy or that were non-responders, remain limited. Gemcitabine (Gem) in combination with *nab*-paclitaxel remains a frequently utilized cytotoxic therapy, as the alternative regimen FOLIFRINOX is difficult to tolerate. Gem is often the only systemic therapy that is tolerated by patients that are more frail and with more advanced disease 3. Chemoresistance can be related to changes in the tumor environment, drug metabolism and drug efflux 4. Metabolic reprogramming, a known mechanism of chemoresistance leading to cancer cell proliferation and survival, can arise in response to genotoxic stress 5. How genotoxic stress leads to metabolic reprogramming has not been clearly defined. Genotoxic chemotherapy agents can induce the *de novo* pyrimidine synthesis pathway to increase the availability of nucleotides essential for DNA repair 6. Inhibition of the pyrimidine synthesis pathway can sensitize cancer cells to genotoxic chemotherapy agents 7. Leflunomide (LEF), an agent with a long history of safety and efficacy in the treatment and prevention of autoimmune disorders and allograft rejection, targets *de novo* pyrimidine synthesis via inhibition of dihydroorotate dehydrogenase (DHODH) 8, 9. DHODH is the rate-limiting enzyme in the synthesis chain of uridine and is a critical enzyme in this pathway 10. Although LEF has also been shown to modulate T cell responses in autoimmune diseases, the role of LEF on the immune microenvironment in solid tumors has not been clearly defined. Pre-clinical data show that LEF has potent anti-neoplastic effects in multiple myeloma, oral squamous cell carcinoma, renal cell carcinoma, melanoma and non-small cell carcinoma 11-15. In this study we aim to assess the effect of LEF with Gem in an immune competent model of pancreatic cancer and to study the immune tumor microenvironment and changes in *de novo* pyrimidine biosynthesis.

## Methods

### Cell culture and Animal Model

The KPC PDAC cell line was obtained from Ximbio (UK). Briefly, this cell line was derived from pancreatic tumors of LSL-**K**ras-G12D, LSL-**p**53-R172H, and Pdx-1-**C**re mice (KPC) mice. KPC cells were grown at 37 °C, 5% CO_2_ atmosphere in complete endotoxin-free DMEM media (Corning, USA) containing high glucose and glutamine supplemented with 10% FBS (Gibco, USA) and 1% penicillin and streptomycin (Gibco, USA). 6-week-old female C57BL6 mice were purchased from Jackson Lab and maintained under specific pathogen-free conditions at the City of Hope (COH) Animal Research Center. Animals were handled according to Institutional Animal Care and Use Committee (IACUC) guidelines under the approved protocol #16067.

### Reagents

Gemcitabine (G4177500MG, LC Laboratories) was reconstituted in water or 0.9% saline for *in vitro* or *in vivo* experiments, respectively. The drug was freshly made and injected intraperitoneally into the mice at the dose of 75 mg/kg. Teriflunomide (S4169, Selleckchem) and Leflunomide (J65917ME, Alfa Aesar) were reconstituted in DMSO or CMC/Tween 80 for *in vitro* or *in vivo* experiment, respectively. On the day of treatment, the drug was prepared and administered via oral gavage into the mice at a dose of 40 mg/kg.

### Cell proliferation assay and Combination experiment

The growth inhibition of the KPC cell line was determined using the 3-(4,5-diethylthiazol-2-yl)-5-(3-carboxymethoxyphenyl)-2-(4-sulfopheyl)-2H-tetrazolium (MTS) assay (CellTiter 96 Aqueous One Solution, Promega, USA). Cells (1×10^4^ cells/100 µl/well) were seeded in 96-well tissue culture plates. After 24hrs, cells were incubated with Teriflunomide (2.5-80 µM) and/or Gem (2.5-80 nM) as single agents or in combination at a constant ratio of Teriflunomide:Gem = 1000:1. After 72 hrs of treatment, cell proliferation was evaluated using an MTS assay according to the manufacturer's instructions. Briefly, 20 μl of MTS reagent was added to each well, and the plates were incubated for 2hrs. Absorbance at 492 nm was measured with a microplate reader (Filtermax F3). Results were represented as the means ± standard deviation of the mean (SD) from triplicate wells. For potential synergistic or additive effects in a drug combination, combination index (CI) values were calculated using CompuSyn software (Cambridge, UK). In order to assess potential synergy or additive effects in drug combinations, the CI is calculated by Chou-Talalay method 16. This provides the theoretical basis for the CI-isobologram equation that allows quantitative determination of drug interactions. Based on these algorithms, Compusyn software by Chou was applied to assed the drug synergism, addition and antagonism effects at ED50, ED75, ED90 and ED95; which are the effective doses (EDs) at which 50, 75, 90 and 95% of cells are killed. Drug synergism, addition and antagonism effects are defined by CI values of <1.0, 1.0 and >1.0 respectively.

### Tumor challenge and therapy

KPC cells (5 × 10^5^) were suspended in PBS and implanted subcutaneously into the right thigh of C57BL6 mice. Three weeks after tumor inoculation, when the tumor sizes reached 50 mm^3^, the mice were randomized into 4 groups (*n*=5/group): control, Gem, Leflunomide, and combination. The mice were treated in the following dosing regimens for 4 weeks: (i) intraperitoneal injection of Gem (75 mg/kg) every three days, and (ii) oral gavage of Leflunomide (40 mg/kg) every day. Tumor volume (mm^3^) was measured once a week with a caliper until the tumor volume exceeded 1000 mm^3^ or any experimental endpoint, as pre-determined in the IACUC protocol, was reached (V=1/2 × Length × Width × Depth).

### Preparation of single cell suspensions from tumor tissues and peripheral blood

At day 28 after the first treatment, animals were euthanized, tumors and blood were harvested. The collected tumor tissues were washed with PBS and minced into 2-3 mm^3^ pieces using sterile blades. The tissue fragments were then incubated with Collagenase IV (1 mg/ml) and DNAse I (50 ug/ml) for 20 minutes at 37 °C followed by an incubation on ice for 10 minutes to stop the enzymatic digestion. The digested tissue fragments were gently homogenized using a plunger from a 1 ml syringe in serum-free RPMI (Corning, USA), followed by dissociation using a 40 μm cell strainer. The collected tumor cells were washed and re-suspended in PBS prior to further staining. Red blood cells were lysed using RBC lysis buffer (Biolegend, USA). Peripheral blood mononuclear cells (PBMCs) were collected, washed and re-suspended in PBS for further manipulation.

### Flow cytometry analysis

For surface staining, single cell suspensions from tumors and PBMCs (1×10^6^) were prepared as described above and incubated with 1 μl Fixable Viability Dye eFlour 506 for 10 min on ice. Next, cells were washed with PBS and incubated with fluorescence-labeled antibodies against target cell surface molecules for 30 minutes in the dark on ice. Next, for intracellular staining, cells were fixed and permeabilized using Fixation/Permeabilization solution (Invitrogen, USA) and then block with anti-mouse CD16/CD32. Cells were stained with intracellular markers (FoxP3 or CTLA-4) in permeabilization buffer for 1 hour. Four different flow cytometry panels have been applied in this study: T cell activation panel using CD3 PerCP-Cy5.5 (45-2C11), CD4 eFlour450 (RM4-5), CD8 FITC (53-6.7), CD69 APC (H1.2F3); T cell exhaustion panel using CD3 PerCP-Cy5.5 (45-2C11), CD4 eFlour450 (RM4-5), CD8 FITC (53-6.7), CTLA-4 PE (UC10-4B9); Treg panel using CD3 PerCP-Cy5.5 (45-2C11), CD4 FITC (RM4-5), CD25 APC (PC61.5), FoxP3 PE (FJK-16s); and MDSC panel using CD45-eFlour450 (30-F11), CD11b PE (M1/70), Ly6G FITC (1A8), Ly6C APC (HK1.4). All labeled antibodies were purchased from eBioscience (USA). At least 10,000 events were analyzed using a FACS Celesta flow cytometer (BD), according to the manufacturer's instructions. Doublets were excluded and alive cells were used for evaluation using FlowJo software (TreeStar).

### Immunohistochemistry staining

For immunohistochemistry (IHC) staining, excised tumors from mice were fixed in 10% Neutral Buffered Formalin (NBF), processed and embedded in paraffin. Tumor blocks were then sectioned at a thickness of 5 μm and put on positively charged glass slides. IHC staining was performed on Ventana Discovery Ultra (Ventana Medical Systems, Roche Diagnostics, Indianapolis, USA) IHC automated stainer. The slides were loaded on the machine; deparaffinization, rehydration, endogenous peroxidase activity inhibition and antigen retrieval were first performed. The slides were then incubated with a primary antibody (CD31 and Ki67, Cell Signaling) followed by DISCOVERY anti-Rabbit HQ and DISCOVERY anti-HQ HRP detection system. The IHC tumor sections were visualized with DISCOVERY ChromoMap DAB Kit, counterstained with Hematoxylin (Ventana). The slide images were acquired with Leica Dmi8 Microscope (Leica Microsystems, USA) and analyzed by Image-Pro Premier Software.

### Samples preparation for LC/MS Metabolomics

For *in vitro* experiments, KPC cells were seeded overnight in 6 well-plates using completed DMEM. DMEM with ^13^C/^15^N-isotope labeled nutrients [L-glutamine-(amide-^15^N), Sigma] was prepared to replace ^12^C/^14^N nutrients. Cells were treated with 20 nM Gem and 40 µM Leflunomide as single agents or in combination in isotope tracing medium for 4hrs and 24 hrs. Afterward medium was aspirated, immediately washed with PBS and metabolism was quenched with extraction buffer (40:40:20 acetonitrile:methanol:water with 0.5% formic acid). Plates were placed on dry ice for 10 min and neutralized with 15% NH_4_HCO_3_. Cell lifter was used to scrape cells from plates, extraction buffer containing cells was transferred into Eppendorf tubes and centrifuged in a benchtop microfuge at maximum speed for 30 min at 4 °C. Supernatants were transferred to LC-MS vials for analysis. For *in vivo* experiments, tumor tissues were collected and immediately clamped into liquid nitrogen. Tissues were stored in -80 °C until analysis. Frozen tissues were transferred into precooled 2 mL Eppendorf tubes, and pulverized with a cyromill. Around 10 mg of tissue was weighed, and extraction buffer was added (40 µL extraction buffer per mg tissue). The solution was kept on ice for 10 mins, neutralized with NH_4_HCO_3_ as described above and centrifuged in a microfuge at a maximum speed for 30 min at 4 °C. Supernatants were transferred to LC-MS vials for analysis.

### LC/MS procedures

LC/MS was performed to detect 15N-labeled isotopes of metabolites in the *de novo* pyrimidine synthesis pathway. LC separation was achieved using a Vanquish UHPLC system (Thermo Fisher Scientific) and an Xbridge BEH Amide column (2.1 mm × 150 mm × 2.5 mm particle size, 130 A °pore size; Waters, Milford, MA), column temperature 25°C. Solvent A is 95:5 water:acetonitrile with 20 mM ammonium acetate and 20 mM ammonium hydroxide at pH 9.4, and solvent B is acetonitrile. Flow rate was 150 mL/min. The LC gradient was 0 min, 85% B; 2 min, 85% B; 3 min, 80% B; 5 min, 80% B; 6 min, 75% B; 7 min, 75% B; 8 min, 70% B; 9 min, 70% B; 10 min, 50% B; 12 min, 50% B; 13 min, 25% B; 16 min, 25% B; 18 min, 0% B; 23 min, 0% B; 24 min, 85% B. 25 min, stop run. Injection volume was 5 µL. The Q-Exactive Plus mass spectrometer was operated in negative ion mode scanning from m/z 70-1000 with a resolution at 140,000. Data were analyzed by using El-Maven.

### Statistical Analysis

Statistical analyses were performed using GraphPad Prism 3 Software (San Diego, CA). *P* values were calculated by the Student's t-test or two-way ANOVA and were considered significant if P<0.05. The data were expressed as the mean ± standard deviation (SD) in the figures.

## Results

### Leflunomide enhances the anti-Proliferative Effect of Gemcitabine in KPC cells *in vitro*

**Figure 1:**
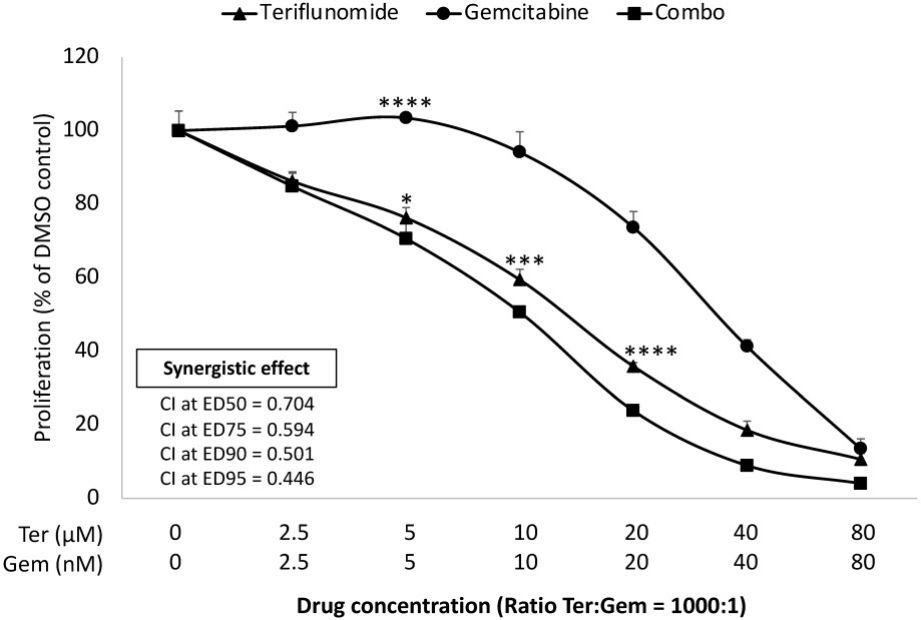
** Leflunomide enhances inhibitory effect in combination with Gem in KPC cell lines *in vitro*.** KPC cells were seeded in 96 well-plate and then treated with Teriflunomide (0-80 µM) and Gem (0-80 nM) for 72 h, as single agents and in combination, at constant ratios (Ter:Gem = 1000:1). MTS assay was performed to measure the cell proliferation. Combination index (CI) values for potential synergistic or additive effects were calculated using CompuSyn software (Cambridge, UK). Drug synergism, addition, and antagonism effects were defined by CI values of <1.0, 1.0, and >1.0, respectively. CI values at ED50, ED75, ED90 and ED95 for combination treatment are shown. Results from one representative experiment are presented as means ± SD, with triplicate determinations. (*) p <0.05, (**) p<0.01, (***) p<0.001, and (****) p< 0.0001 compared to combo.

An established mechanism of Gem resistance in pancreatic cancer patients is the induction of *de novo* pyrimidine synthesis 17. Leflunomide targets the enzyme DHODH which is important in the pathway of *de novo* pyrimidine synthesis. The impact of growth inhibition of leflunomide and Gem was assessed in KPC cells lines *in vitro* using the MTS assay. As Leflunomide *in vivo* is rapidly converted into its active form Teriflunomide (Teri), we used Teri in this assay 18. As shown in **Figure 1**, Teriflunomide alone (2.5-80 µM) suppressed proliferation of KPC cells in a dose-dependent manner. In addition, Teri enhanced the inhibitory effect of Gem (ratio Teri:Gem = 1000:1) in KPC cells (p<0.05 from 0.5 µM Teri and/or 5 nM Gem). Based on the combination index (CI) values of <1, these results indicate that there is demonstrable synergy between Teri and Gem. This data merited further work to be done in the *in vivo* setting. As Lef has been utilized in the treatment of autoimmune disorders, it was important to evaluate the efficacy of Lef and Gem in an immunocompetent *in vivo* model.

### Leflunomide and Gemcitabine suppress tumor growth of KPC cells in an immunocompetent syngeneic flank mouse model

**Figure 2:**
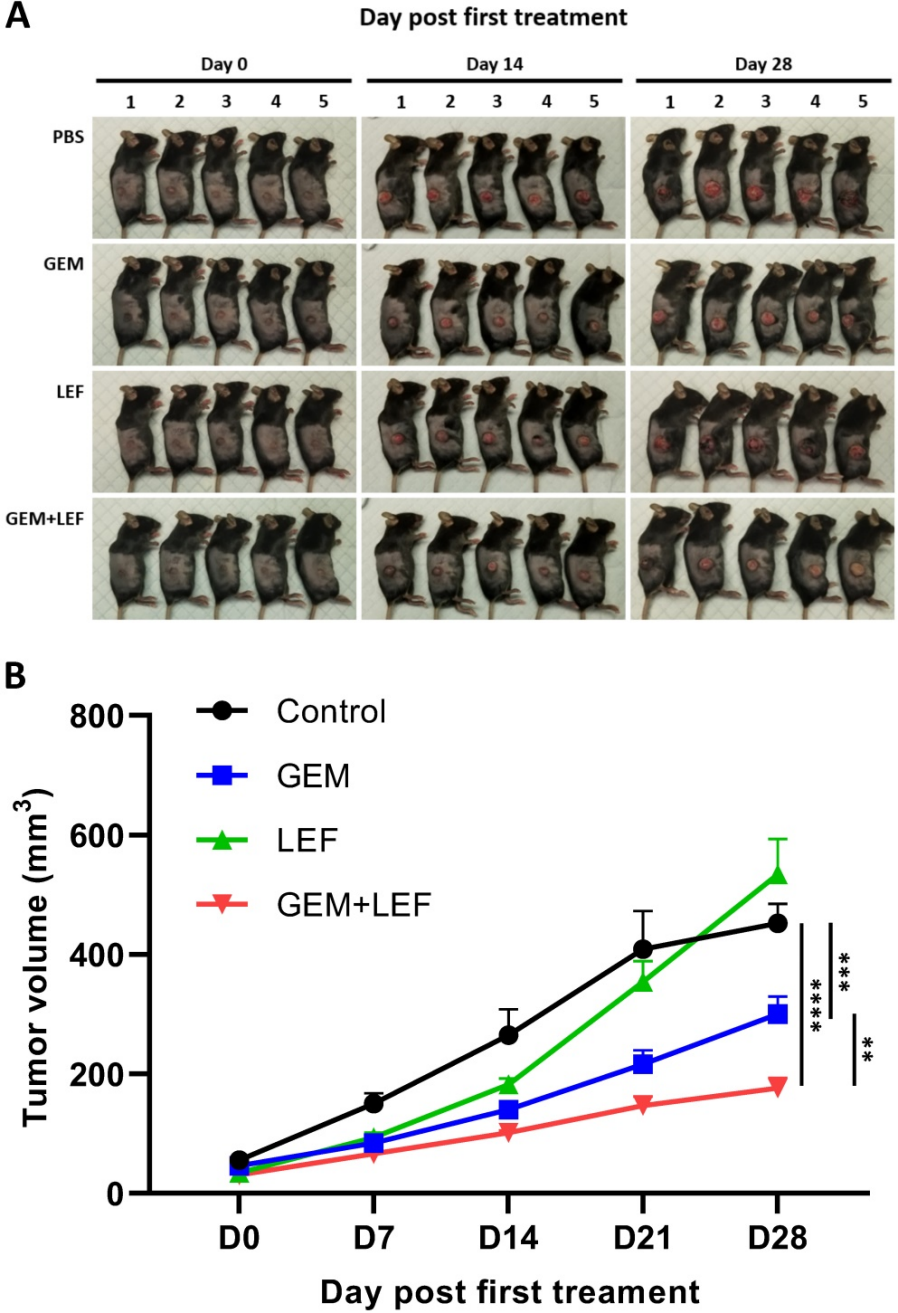
** Combination of Leflunomide and Gem suppress tumors growth using KPC subcutaneous mouse models.** 6-week-old female C57BL6 mice (n = 5/group) were subcutaneously injected into the right thigh with KPC cells (5 × 105 cells/mouse). After 21 days, when the tumor volume reached around 50 mm3, the mice were randomized into 4 groups: control, Gem, Leflunomide, and combination. The mice were treated in the following dosing regimens for 4 weeks: (i) Gem 75mg/kg i.p. every three days, and (ii) Leflunomide 40mg/kg p.o. daily. Tumor volume was measured every 7 days until the end of experiment. Mice were then euthanized when the tumor volume reached 1000mm3. (A) Photos of mice after treatment. (B) Tumor growth curve. Data are presented as means ± SD. (*) p <0.05, (**) p<0.01, and (***) p<0.001 at day 28.

We determined the therapeutic efficacy of Gem, Lef and in combination in a subcutaneous (s.c.) tumor model of KPC cells. When the s.c. tumors reached ≥50 mm^3^, mice were treated with: PBS (control), Gem, Leflunomide, and combination (Gem+Lef) for 4 weeks (**Fig. 2**). Although leflunomide attenuated tumor growth from day 7 to day 14, statistical significance was not reached. Similar to early preclinical trials using Gem and Lef in an immunosuppressed mouse model, Gem and combination Gem+Lef significantly halted tumor progression 19. There was significant growth inhibition by day 21 and persisting through day 28 in Gem+Lef treatment (p<0.05 at day 28). These results report for the first time that Lef (an agent used to mitigate autoimmune conditions) in combination with Gem has anti-tumor growth abilities in an immunocompetent flank model of pancreatic cancer.

### Immune cell profiling of Leflunomide and Gemcitabine treatment in KPC subcutaneous mouse models

**Figure 3:**
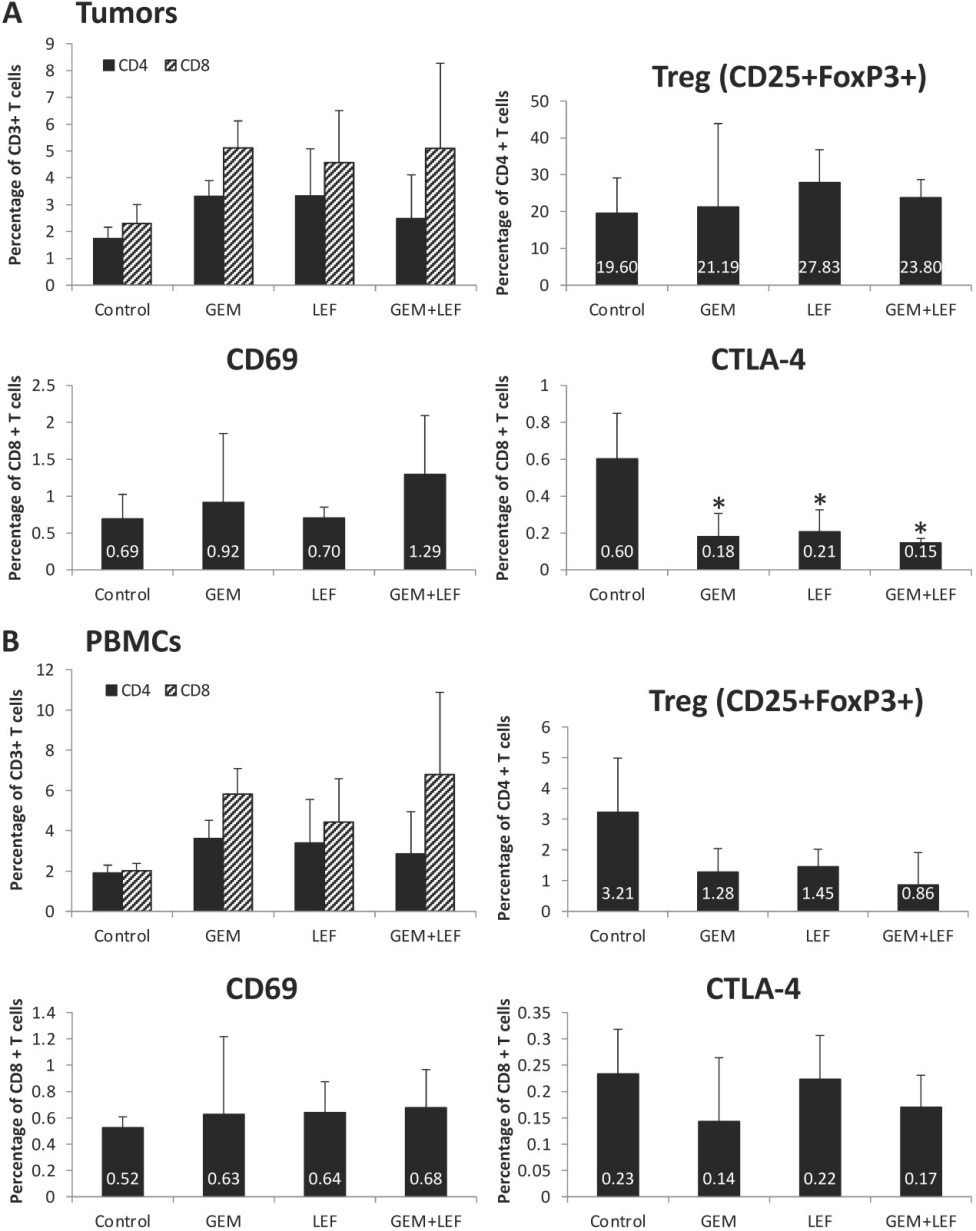
** T cell profiling of Leflunomide and Gem treatment in KPC subcutaneous mouse models.** KPC subcutaneous mouse models (50mm^3^) in C57BL6 mice were treated with Gem, Leflunomide as single agents or in combination as described in the legend of figure 2. (A) At day 28 after the first treatment, tumors were excised and single-cell suspensions were prepared. Cells were then stained with different cell markers CD3, CD4, CD8, CD25, FoxP3, CD69, and CTLA-4. Percentage of CD4+T cells, CD8+T cells from total CD3+ T cells; (CD4+CD25+FoxP3+) Treg cells, (CD8+CD69+) activated T cells, (CD8+CTLA-4+) suppressive T cells from total CD8+ T cells were analyzed using flow cytometry. (B) At day 28 after the first treatment, bloods were collected and PBMC were extracted for further staining. Same procedures were performed for FACS analysis of T cells in PBMCs. Data are presented as means ± SD. (*) p <0.05 compared to control.

In order to assess the role of the immune response in inducing remission following Gem+Lef treatment and as Lef is known to induce changes in the immune cell populations, we investigated the immune profile in treated tumors and PBMCs. First, we sought to examine changes in T cell populations in tumors after 28 days of treatment. As shown in **Figure 3A**, we first looked at CD4 and CD8 cell populations. There was a significant decrease in expression of markers of T cell exhaustion. All the treated (Gem, Lef, Gem+Lef) tumors showed marked down regulation in the T cell exhaustion marker (CTLA-4+) compared to controls (p<0.05). We chose to look at CTLA-4+ as a marker of exhaustion as uur group has previously studied Leflunomide in multiple myeloma 5TGM1 model in C57BL/KaLwRijHsd mice, which showed significantly differences in only CTLA-4 CD8+ T cells but not other markers of T cell exhaustion (LAG3, PD1, 2B4 and KLRG1) 18.

In order to assess changes in the circulating blood, PBMCs were evaluated for populations of T cells. There were no significant changes in markers of T cell activation (CD69+ T cells) or markers of T cell exhaustion (CTLA4+ T cells) based on the treatment group in the PBMC populations (**Fig. 3B**).

**Figure 4:**
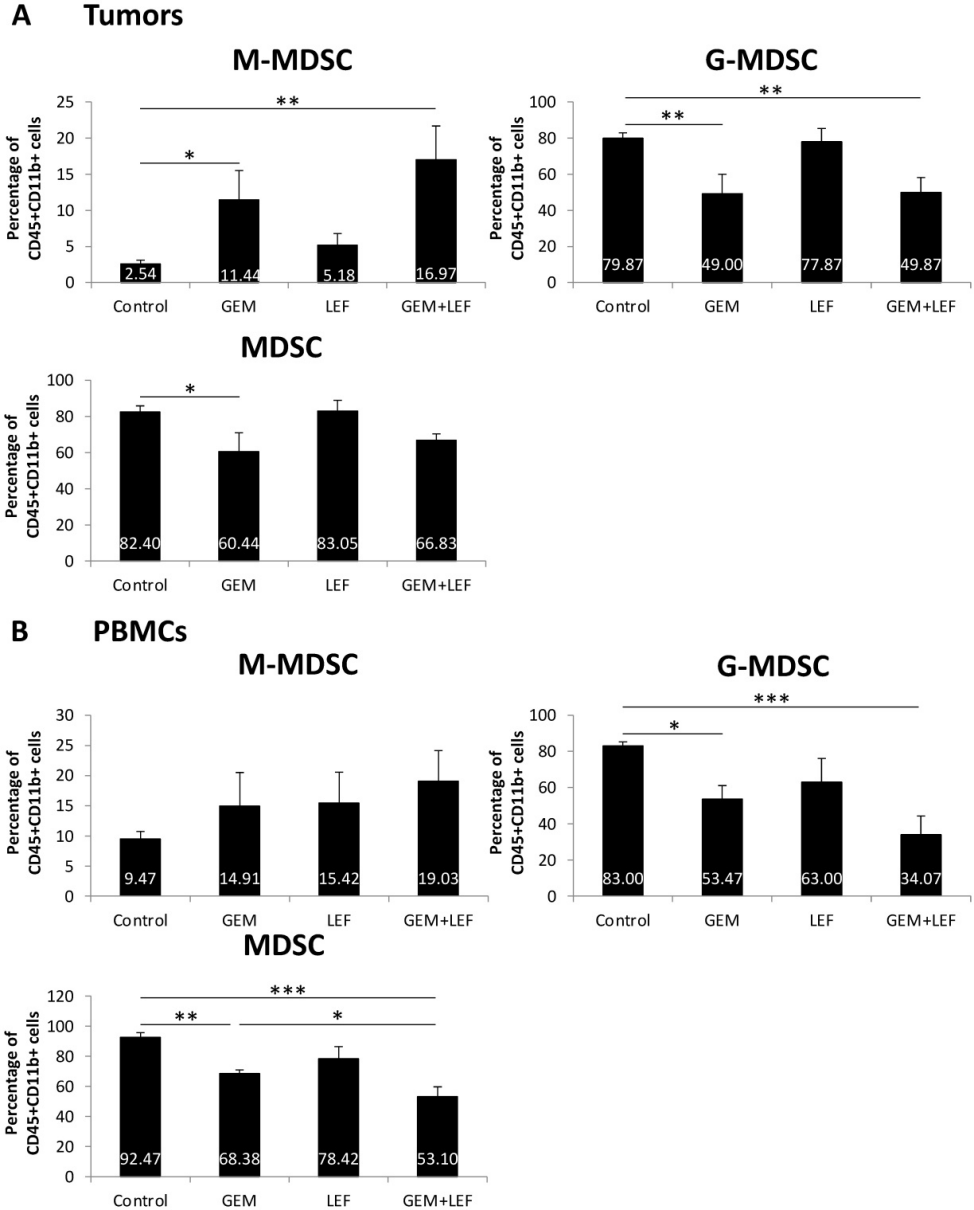
** Myeloid-Derived Suppressor Cells (MDSC) populations of Leflunomide and Gem treatment in KPC subcutaneous mouse models.** On the same experiment as mentioned in figure 3, single-cell suspension from tumors (A) and PBMCs (B) at day 28 after the first treatment were applied for staining with other cell markers for MDSCs: CD45, CD11b, Ly6G and Ly6C. Percentage of (CD11b^+^Ly6G^-^Ly6C^hi^) monocytic (M-MDSC) and (CD11b^+^Ly6G^+^Ly6C^lo^) granulocytic or polymorphonuclear (G-MDSC) from total CD45+CD11b+ cells were analyzed using flow cytometry. Data are presented as means ± SD. (*) p <0.05, (**) p<0.01, (***) p<0.001.

As myeloid-derived suppressor cells (MDSCs) are important in a variety of mechanisms that alter T cell activation or suppression, we studied the population of MDSCs in tumors and in PBMCs in mice treated with Gem, Lef, or Gem+Lef and controls (**Fig. 4**). We looked at both monocytic-MDSCs (M-MDSCs) and the granulocytic fraction (called granulocytic/polymorphonuclear MDSCs or G-MDSCs). The M-MDSCs are thought to have more immunosuppressive activity.^20^, ^21^ In the tumor models, we found that treatment with Gem alone and Gem+LeF significantly increased the population of M-MDSCs, indicating an increase in the immunosuppressive environment (**Fig. 4A**, (p<0.05). The G-MDSCs decreased in the treated groups of Gem (p<0.01) and Gem+Lef (p<0.01) but not in Lef alone compared to controls. As there was a greater proportion of G-MDSCs, the overall changes of MDSCs in the tumors were only significantly decreased with Gem alone (p<0.05). In the PBMCs, the findings were similar with a significant decrease in G-MDSCs in the Gem and Gem+Lef treated groups compared to controls (**Fig. 4B**, p<0.05). Like the tumors there was a significant decrease in overall MDSCs in the Gem and Gem+Lef groups compared to controls (p<0.01 and p<0.001).

The immune profiling results in the tumors and in the PBMCs can be summarized to indicate that Lef may be enhancing the anti-tumor immunity of Gem treatment via a mechanism that favors the recruitment of activated CD69+CD8+ T cells in the tumors while decreasing the frequency of Tregs, M-MDSCs (systemically) and CTLA-4+CD8+ T cells in the tumor microenvironment. Altogether, Gem+Lef treatment appears to tilt the balance of the host from an immunosuppressive to an immune activated state, thus further delaying tumor progression.

### Leflunomide causes anti-angiogenesis effect and enhances the anti-tumor effect of Gemcitabine in KPC subcutaneous mouse models

**Figure 5:**
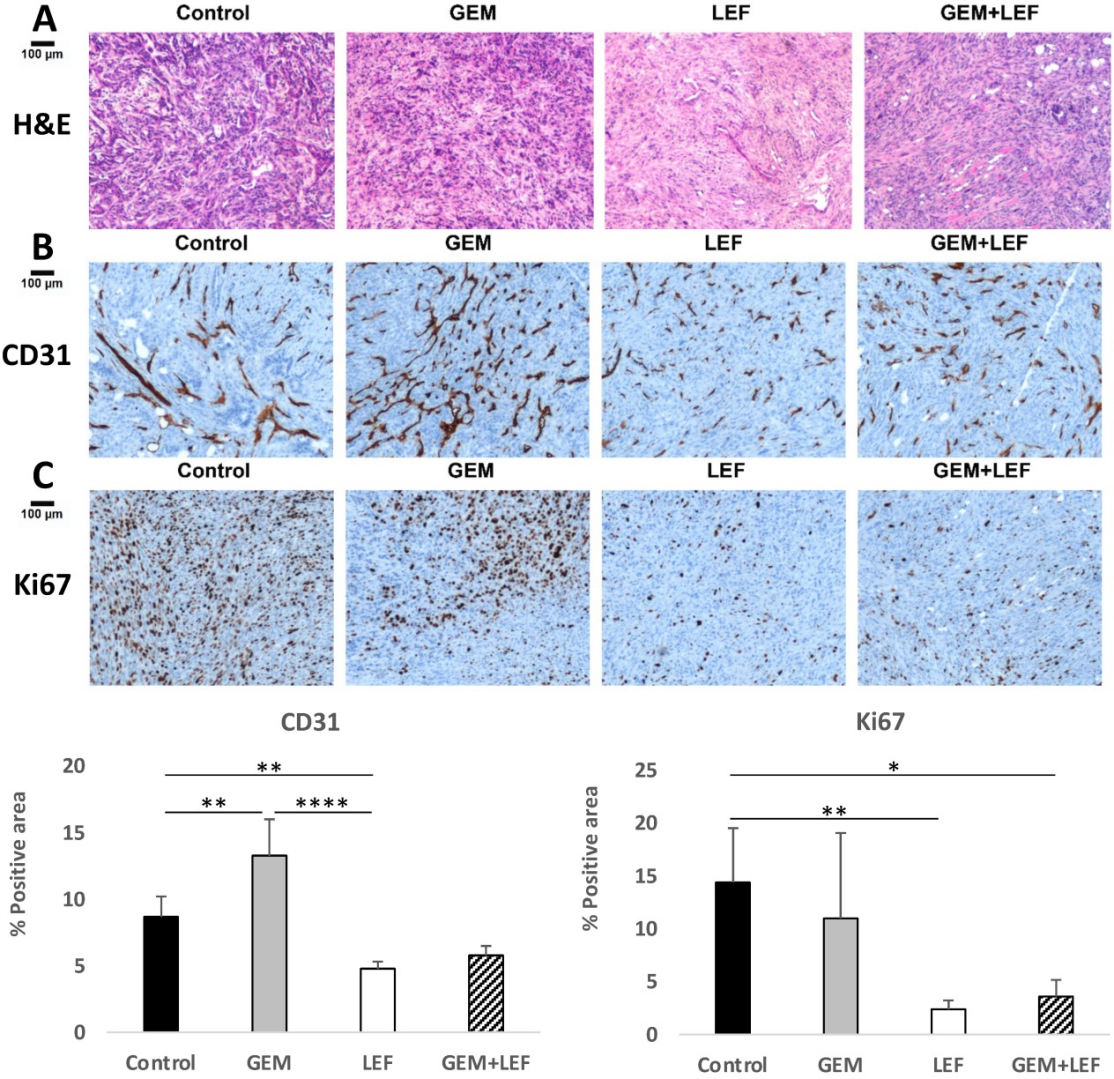
** Leflunomide causes anti-angiogenesis effect and enhances the anti-tumor of Gem in KPC subcutaneous mouse models.** KPC subcutaneous mouse models (50 mm^3^) in C57BL6 mice were treated with Gem, Leflunomide as single agents or in combination as described in the legend of figure 2. At day 28 after the first treatment, tumors were excised and immunohistochemistry (IHC) staining was performed with specific antibodies: (B) CD31 (microvessel density) and (C) Ki67 (cell proliferation). Representative photography of H&E (A) and IHC staining of KPC tumor tissues from different experimental groups are shown. Positive staining appears as brown color. Protein expression levels were analyzed by calculating the percentage of integrated optical density (IOD)/area using Image-Pro Premier. Data are presented as means ± SD. (*) p <0.05, (**) p<0.01, and (***) p<0.001.

Based on data that Lef has been shown to inhibit angiogenesis in other solid tumors, CD31staining was performed to evaluate microvessel density. Explanted tumors from mice with flank tumors that had been treated with Gem, Lef, Gem+Lef were stained for CD31 and compared to controls (**Fig. 5B**). The results for CD31 staining indicate that both the Lef alone and Gem+Lef tumors had significantly less CD31 positive staining indicating inhibition of angiogenesis (P<0.01).

In order to assess if inhibition of angiogenesis also correlated to growth inhibition, tumors were also stained for the proliferation index marker Ki-67. As shown in **Figure 5C**, Lef alone or Gem+Lef treatment led to significantly fewer proliferating cells (Ki67+ cells) in KPC tumors (P<0.01). These findings indicate that Lef alone and in combination with Gem resulted in both inhibition of angiogenesis and proliferation in this KPC flank model of pancreatic cancer.

### Leflunomide inhibits *de novo* pyrimidine synthesis in KPC cells both *in vitro* and *in vivo*

**Figure 6:**
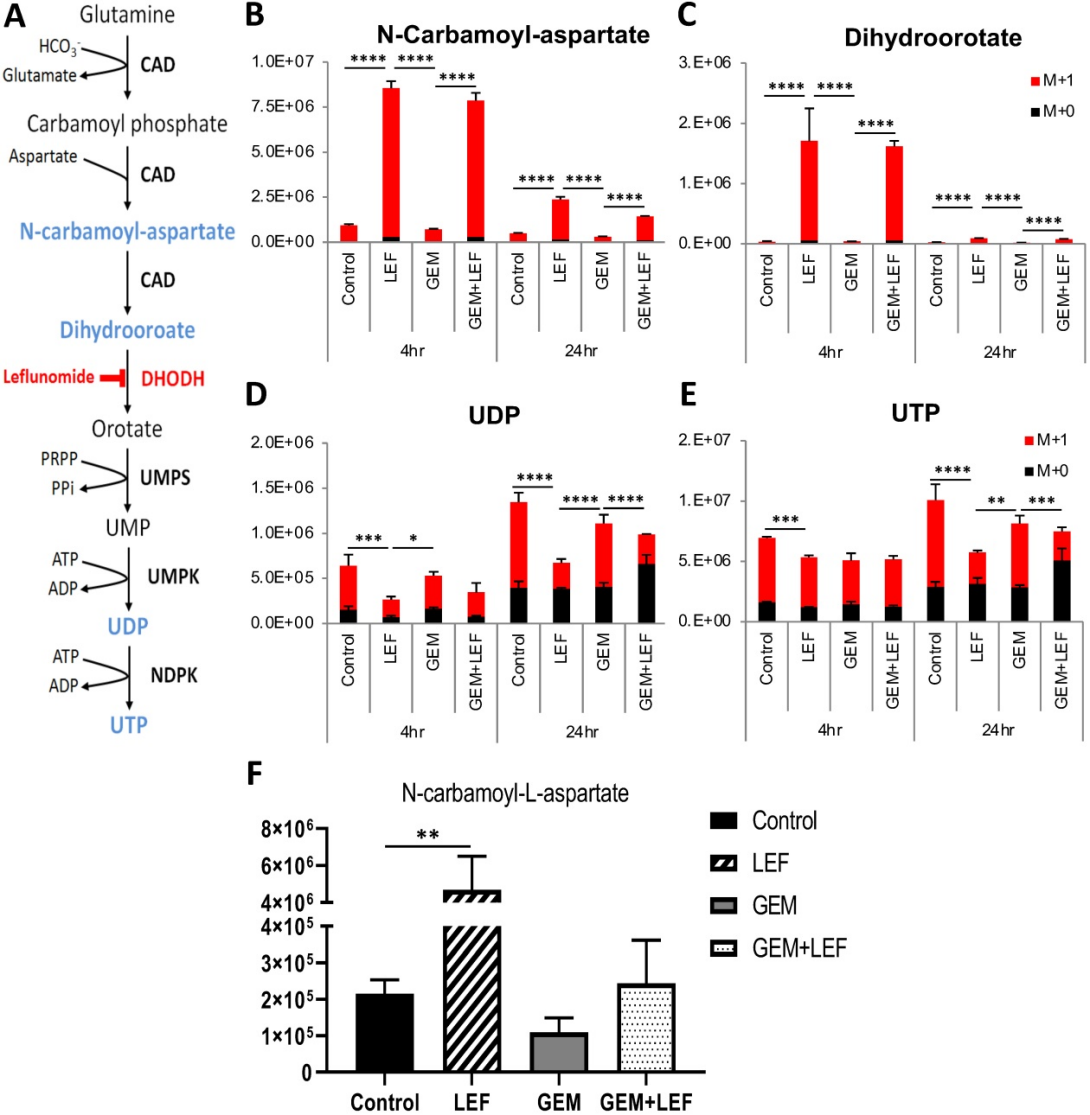
** Leflunomide inhibits the de novo pyrimidine synthesis pathway in KPC cells *in vitro* and *in vivo*.** (A) Schematic of the de novo pyrimidine synthesis pathway. KPC cells were treated with Gem 20nM, Leflunomide 40μM as single agents or combination in DMEM supplemented with 200 μM L-Glutamine-(amide-^15^N), 10% FBS and 1% Penicillin-Streptomycin. After 4hr and 24hr of treatment, cells were quenched with ice-cold solvent (40:40:20 Acetonitrile:Methanol:Water with 0.5% formic acid) and cellular metabolites were extracted. Relative isotopic enrichment of M L-Glutamine-(amide-^15^N) into Dihydroorotate (B), Carbamoyl-aspartate (C), UDP (D) and UTP (E) was measured by LC/MS-MS. (F) Level of Carbamoyl-aspartate from tumor tissues. Results from one representative experiment are presented as means ± SD, with triplicate determinations. (*) p < 0.05, (**) p < 0.005, (***) p < 0.001, and (****) p < 0.0001 by two-way ANOVA. M+0, all amides unlabeled ^14^N amide and M+1, all amides labeled ^15^N.

Leflunomide and its active form Teriflunomide (Teri) have been known as a selective inhibitors of the *de novo* pyrimidine synthesis pathway. Lef inhibits the rate-limiting enzyme DHODH (**Fig. 6A**). The anti-proliferative effect of Gem+Teri on KPC cells is demonstrated in **Figure 1.** These findings suggest that Teri enhances the antitumor activity of Gem through the depletion of the nucleoside precursors required for DNA damage repair. To confirm this hypothesis, LC/MS was performed to detect 15^N^-labeled isotopes of metabolites in the *de novo* pyrimidine synthesis pathway in KPC cells. The *in vitro* results showed that Teri alone and Gem+Teri combination therapy significantly increased the abundance of metabolites upstream of DHODH; specifically, there were increased levels of *N*-carbamoyl-aspartate and dihydroorotate after 4 hrs and 24 hrs of treatment (**Fig. 6B & C**) p<0.001. Consistently, decreased levels of UDP and UTP were observed in Teri and Gem+Teri combination treated cells (**Fig. 6D & E**). The *in vivo* findings were similar. After 28 days of treatment, Lef induced inhibition of the DHODH enzyme was demonstrated by changes in measured metabolites. The metabolomics profile of Lef treated tumors yielded a significant increase in the proportion of *N*-Carbamoyl-aspartate when compared with the controls or Gem treated tumors (**Fig. 6F**) p<0.01. Our findings are consistent in that with Lef treatment there is significant inhibition of *de novo* pyrimidine synthesis in both *in vitro* and *in vivo* models and this may account for in part the mechanism of growth inhibition seen in Gem+Lef combination therapy.

## Discussion

In this study we demonstrate for the first time that Gem + Lef inhibits tumor growth in part via inhibition of angiogenesis, proliferation, *de novo* pyrimidine synthesis and induces a favorable anti-tumoral immune phenotype in an immunocompetent mouse model.

Pancreatic cancer remains a challenging disease to treat. Patients are often not robust enough to tolerate the more aggressive regimens of Gem with *nab*-paclitaxel or FOLFIRINOX 22. Therefore, Gem alone remains a mainstay in systemic therapy. Unfortunately, we know from randomized controlled trials that after several months of therapy, tumor resistance develops and cancer progression ensues 23. There are several described mechanisms of Gem resistance, which involve the desmoplastic tumor microenvironment, changes in Gem transport, changes in the enzymatic activation of Gem and a host of associated intracellular enzymes and transcription factors 24, 25. One particular mechanism of Gem resistance is *de novo* pyrimidine synthesis 19. Pyrimidine bases are critical for cellular metabolism and growth and are important precursors in DNA (thymine and cytosine) and RNA (uracil and cytosine) biosynthesis 26. Lef targets the enzyme DHODH which is a rate limiting step in *de novo* pyrimidine synthesis 27. Lef binds reversibly to the alpha-helical domain of DHODH and blocks access to this active site inhibiting enzyme activity 27. Lef has been studied in multiple solid and liquid tumors as an antiproliferative agent 27. In triple negative breast cancer, inhibition of *de novo* pyrimidine synthesis sensitizes cells to genotoxic chemotherapy agents by exacerbating DNA damage 7. Combining treatment with doxorubicin and leflunomide induces regression of TNBC xenografts.

Based on the mechanism of action, the combination of Lef and Gem in an immunocompetent model of pancreatic cancer was the next step in moving this potential therapy to patients. We first evaluated the potential synergy of Gem and Teriflunomide (the active *in vitro* metabolite Lef) in the KPC mouse pancreatic cell line. Lef is an isoxazole derivative (N-[4-trifluoromethylphenyl]-methylisoxazol-4-carboxamide) that is an oral prodrug that is hydrolyzed during first-pass metabolism in the gut and liver to its singe active metabolite teriflunomide) 26. The therapeutic effects of leflunomide are primarily mediated via this metabolite 28. Similar to our published *in vitro* studies in human pancreatic cancer cell lines, we were able to demonstrate synergy between Gem and Teri and this prompted our *in vivo* work 29. As KPC mice harbor p53 and Kras mutations, they have been studied as a well-accepted model in performing preclinical work in the field 30. Additionally, as Lef is in part an immunomodulator, it was important to perform the *in vivo* work in an immunocompetent syngeneic model.

Our *in vivo* findings demonstrate that Gem and Lef together significantly induce growth inhibition of flank implanted KPC cell tumors. The combination of Gem and Lef have been previously studied and demonstrated the synergy with human pancreatic cancer cells implanted in an immunocompromised mouse and in KPC mice 19, 31. Yu *et al* demonstrated that mitochondrial fusion was a potential target/regulator of pancreatic cancer growth. PDAC requires mitochondrial oxidative phosphorylation (OXPHOS) for growth. They demonstrated that oral leflunomide promoted mitochondrial fusion which reduced OXPHOS and thus inhibited tumor growth 31. Shukla *et al* demonstrated that Gem-resistant cells stabilized hypoxia inducible factor-1 alpha and this lead to increased glycolysis, leading to rapid generation of biosynthetic intermediates to supply the ingredients for cell growth and proliferation 19. They demonstrated that inhibition of DHODH a key enzyme in the pyrimidine biosynthesis pathway with leflunomide, increased Gem sensitivity in the Gem-R cells 19. Their findings in conjunction with our *in vivo* work led us to evaluate the role of Gem+Lef in an immunocompetent model.

Lef is an agent with a long track record of safety that has been used in autoimmune disorders such as rheumatoid arthritis 8. In autoimmune disease, Lef aims to mitigate the immune response. As inflammation in the tumor microenvironment is an important component of tumor progression, the role of Lef as an immunomodulator has not been adequately assessed 32. In this study we demonstrate that Lef treated tumors demonstrated decreased CTLA-4 + T cells indicating a decrease in intratumoral T cell exhaustion and perhaps more anti-tumor immunity. Interesting, the combination of Gem+Lef correlated with an increase in the population of the M-MDSCs which are thought to have more immunosuppressive activity. Inversely, G-MDSCs population decreased in the Gem+Lef groups. These immune changes in the tumor microenvironment are potential explanations for the growth inhibitory changes induced by treatment with Gem+Lef and will be further studied in future work.

Angiogenesis in pancreatic cancer is a debated question. There is evidence that the desmoplasia in these tumors can be protective and mitigate more aggressive spread 33. In contrast, there is literature to say that inhibition of angiogenesis precludes nutrient delivery to tumors and thus can mitigate tumor growth and induce regression 34. We further examined the tumors for markers of angiogenesis as Lef has been shown to inhibit angiogenesis in other solid tumors (bladder cancer). We found similar findings in our Lef treated tumors with significantly less CD31 staining indicating inhibition of angiogenesis. As the blood supply is a route of nutrition for these tumors, inhibition of angiogenesis is likely a relevant mechanism in Gem+ Lef induced growth inhibition in our PDAC model. The proliferation index was also markedly reduced with the combination of Gem and Lef in these tumors and correlating to the *in vivo* gross tumor measurement findings.

Lastly, we sought to examine the metabolomics as they pertained to the mechanism of action of Lef. As noted above, DHODH is a rate limiting enzyme in the *de novo* pyrimidine synthesis pathway. We and others have shown that Lef inhibits this enzyme in human pancreatic cancer cell lines. Here we demonstrate for the first time that Lef inhibits DHODH as measured by upstream metabolite accumulation both *in vitro* and *in vivo* in a KPC syngeneic immunocompetent model of pancreatic cancer.

Based on the above evidence, LEF is active in both metabolic and immune contexts. Further work will need to elucidate which mechanism accounts for a greater magnitude of the effect of LEF on pancreatic cancer growth. It is likely that there is significant overlap with inhibition of DHODH impacting both mechanisms.

## Limitations

One consideration of utilizing Lef with Gem or with alternative regimens is that in the *in vitro* and *in vivo* setting we aren't able to precisely recapitulate the disease process in patients. The thought is that over time *de novo* pyrimidine synthesis is a mechanism of resistance that develops and that Lef may be used as a maintenance therapy option after a period of time on Gem. Due to the pace of disease progression in these mouse models, it is challenging to recreate this scenario. This will be a focus of study in future work.

## Conclusions

In conclusion, our data demonstrates that the combination of Gem and Lef inhibits pancreatic cancer cell growth both *in vitro* and in an immunocompetent *w* model. We were also able to demonstrate that combination therapy is associated with decreased CD8+ T cell exhaustion, angiogenesis and proliferation. We were also able to demonstrate that combination therapy changes the metabolomics of cells *in vitro* and an *in vivo* such that *de novo* pyrimidine synthesis with accumulation of upstream metabolites. This may be a mechanism to leverage in treating patients with pancreatic cancer that develop resistance to Gem therapy without significant added toxicity.

## Abbreviations

Lef: leflunomide
Gem: Gemcitabine
MTS: (3-(4,5-dimethylthiazol-2-yl)-5-(3-carboxymethoxyphenyl)-2-(4-sulfophenyl)-2*H*-tetrazolium
KPC: cell line derived from LSL-**K**RAS-G12, LSL-**p**53-R172H, and PDx-1-**C**re mice)
PBS: Phosphate Buffered Solution
FACS: Fluorescence-activated cell sorting (FACS)
PDAC: pancreatic ductal adenocarcinoma
FOLFIRINOX: Leucovorin, 5-fluorouracil, Irinotecan, Oxaliplatin
DNA: Deoxyribonucleic acid
DHODH: dihydroorotate dehydrogenase
DMEM: Dulbecco's Modified Eagle Medium
COH: City of Hope
IACUC: Institutional Animal Care and Use Committee
SD: standard deviation
CI: Combination Index
RPMI: Roswell Park Memorial Institute Medium
RBC: red blood cell
PBMC: peripheral blood mononuclear cells
IHC: immunohistochemistry
NBF: neutral buffered formalin
LC/MS: liquid chromatography/mass spectrometry
Teri: Teriflunomide
s.c.: subcutaneous
MDSC: myeloid-derived suppressor cells
M-MDSC: monocytic myeloid-derived suppressor cells
G-MDSC: granulocytic myeloid-derived suppressor cells
RNA: ribonucleic acid
TNBC: triple negative breast cancer
OXPHOS: oxidative phosphorylation
